# Marker assisted improvement of low soil phosphorus tolerance in the bacterial blight resistant, fine-grain type rice variety, Improved Samba Mahsuri

**DOI:** 10.1038/s41598-020-78186-5

**Published:** 2020-12-03

**Authors:** H. K. Mahadeva Swamy, M. Anila, R. R. Kale, G. Rekha, V. P. Bhadana, M. S. Anantha, P. Brajendra, C. H. Balachiranjeevi, S. K. Hajira, B. Laxmi Prasanna, K. Pranathi, T. Dilip, M. B. V. N. Kousik, G. Harika, K. Surekha, R. Mahender Kumar, C. Cheralu, V. Gouri Shankar, G. S. Laha, M. S. Prasad, L. V. Subba Rao, M. S. Madhav, S. M. Balachandran, R. M. Sundaram

**Affiliations:** 1 Crop Improvement Section, ICAR-Indian Institute of Rice Research (IIRR), Rajendranagar, Hyderabad, 500030 India; 2 ICAR-Indian Institute of Agricultural Biotechnology (IIAB), Ranchi, 834010 India; 3 ICAR- Sugarcane Breeding Institute (SBI), Coimbatore, 641007 India; 4 College of Agriculture, PJTSAU, Rajendranagar, Hyderabad, 500030 India

**Keywords:** Genetics, Molecular biology, Plant sciences

## Abstract

Improved-Samba-Mahsuri (ISM), a high-yielding, popular bacterial blight resistant (possessing *Xa21, xa13*, and *xa5*), fine-grain type, low glycemic index rice variety is highly sensitive to low soil phosphorus (P). We have deployed marker-assisted backcross breeding (MABB) approach for targeted transfer of *Pup1, *a major QTL associated with low soil P tolerance, using Swarna as a donor. A new co-dominant marker, K20-1-1, which is specific for *Pup1* was designed and used for foreground selection along with functional markers specific for the bacterial blight resistance genes, *Xa21, xa13*, and *xa5*. A set of 66 polymorphic SSR marker were used for the background selection along with a pair of flanking markers for the recombination selection in backcross derived progenies and in BC_2_F_2_ generation, 12 plants, which are homozygous for *Pup1*, all the three bacterial blight resistance genes and possessing agro-morphological traits equivalent to or better than ISM were selected and selfed to produce BC_2_F_3_s. They were evaluated in plots with low soil P and normal soil P at ICAR-IIRR, Hyderabad for their low soil P tolerance, and bacterial blight resistance and superior lines were advanced to BC_2_F_6_. One of the lines, when tested at multiple locations in India was found promising under both normal as well as low soil P conditions.

## Introduction

Rice, one of the most important staple food crops of the globe, which is being grown in more than 117 countries, provides more than one-fifth of the calories and is consumed by more than 3 billion people. Having the highest area under rice cultivation (43.39 million hectares), one-fourth (22%, i.e. 104.3 million tonnes) of the world’s rice is produced in India, with average productivity of 2.40 tonnes ha^−1^^1^. The crop is adversely affected by numerous biotic and abiotic stress factors. Among the abiotic stresses, drought, salinity, and nutrient deficiency are the most important. Among the nutrients which are deficient in rice soils, phosphorus (P) is one of the most vital macronutrients that is required for proper growth and development of the rice plant and its poor availability in the soil is one of the major yield-limiting factors in rice production^2^. Global demand for P fertilizer is increasing continuously, whereas global commercial phosphate reserves are estimated to be depleted within a few decades^3,4^ and no known substitute for P is available presently^5,6^. In India, the soils are either low (49.3% of soils) or medium (48.8% of soils) in terms of available P^7,8^ and this necessitates the country to import the phosphorus-based fertilizers in a larger scale with 90% dependency^9,10^. It is therefore imperative that one should be ready with alternative solutions for this problem, e.g. better crop residue management, adoption of integrated nutrient management and development of low soil P tolerant rice varieties to manage the low soil P problem. Genetic improvement of the tolerance of rice to P-limiting soils need to be one of the focal areas of research and development in rice to minimize the application of phosphatic fertilizers, which is not only necessary for increasing farmers income by saving on the cost of fertilizers but also to sustain the rice production.

Significant variability is available for P use efficiency/low soil P tolerance among the rice genotypes^11–13^. Identification of *Pup1* QTL, which confers tolerance to low soil P^2^ and the better performance of *Pup1* containing rice lines in different genetic backgrounds in both upland and irrigated conditions^14^ opens the way to improve the rice cultivars for low soil P conditions. The *Pup1* QTL has been fine-mapped^15,16^ and closely linked markers have been developed^18^. Using these markers, *Pup1* has been introgressed through marker-assisted backcross breeding (MABB) into the rice varieties-IR-64, IR-72, Dodokan, Batur, and Situ^14^. Further, *Pup1* has been cloned and the candidate gene underlying the QTL has been identified as Os*PSTOL1*^17^. As *Pup1* is known to improve tolerance to low soil P in both irrigated and upland rice varieties and, availability of closely linked and functional markers for *Pup1,* motivated us to genetically improve the tolerance level of the elite, low P sensitive, high-yielding, bacterial blight resistant, fine-grain type Indian rice variety with low glycemic index, Improved Samba Mahsuri (ISM) through the strategy of MABB.

## Results

### Development of new co-dominant marker for *Pup1* from the CAPS marker K20-1

A multiple sequence alignment of sequence derived from the amplified products of K20-1 of Vandana (Low P tolerant and processing *Pup1*), Improved Samba Mahsuri (Low soil P sensitive and devoid of *Pup1*) along with Kasalath reference sequence corresponding to *Pup1* region and Japonica genome sequence (i.e. Nipponbare sequence) from 12th chromosome of IRGSP pseudomolecule release 5 was done in ClustalW. A 3-bp indel was observed between Vandana and ISM and also between Kasalath and Nipponbare. A new co-dominant marker, named K20-1-1, was designed targeting the conserved regions flanking the indel (Supplementary Fig. 1) using primer3 software. The primer sequence of the newly developed marker and physical positions are given in Table 1; Supplementary Fig. 1. The newly developed marker, produced an amplicon of size 89-bp in the low P tolerant varieties, viz., Kasalath and Vandana, while an amplicon of size 92-bp was amplified in the sensitive rice variety, viz., ISM, when resolved in 8% native PAGE (Supplementary Fig. 1). The newly developed marker, K20-1-1 was tested in a F_2_ population consisting of 135 individuals derived from the cross Improved Samba Mahsuri/Swarna. The marker showed perfect co-segregation with trait phenotype, wherein all the 103 tolerant F_2_ plants were observed to amplify the tolerant allele with respect to the newly developed marker in either homozygous or heterozygous condition and all the sensitive F_2_ plants (n = 32) amplified the fragment which is specific for the sensitive allele (Supplementary Fig. 2).

**Figure 1:**
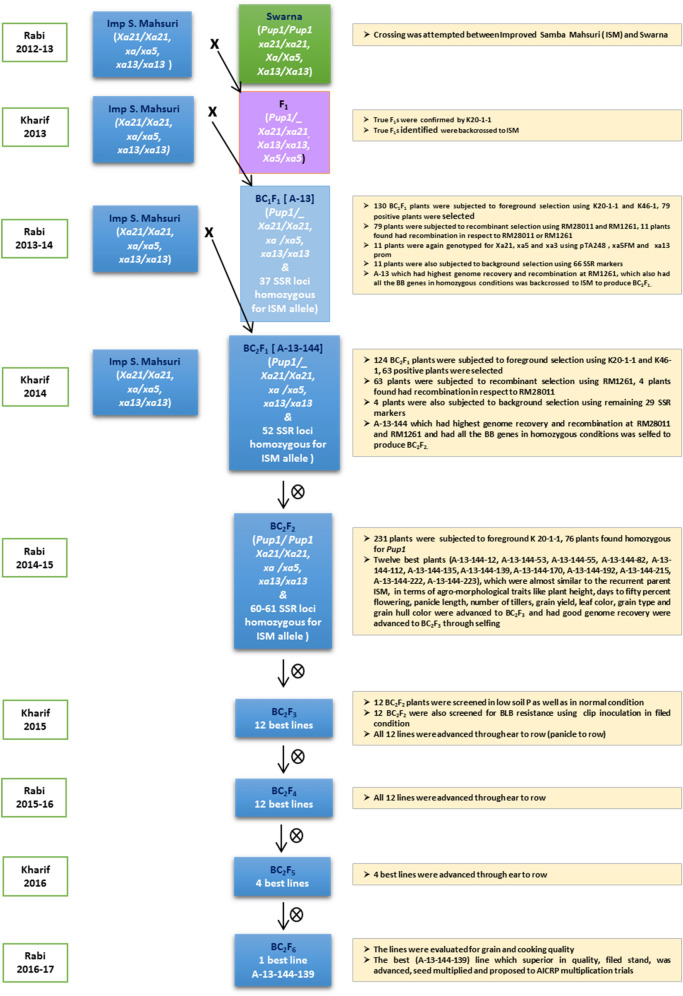
Scheme of MABB followed in the development of ISM-*Pup1* NILs.

**Table 1:** Primer sequences and physical positions of markers K20-1 and K20-1-1.

Primer name	Primer sequence		Amplicon size		Physical location (Mb) in Kasalath AB458444.1	Physical Location (Mb) in Nipponbare (Japonica), 12th Chromosome
			K	N		
K20-1	F	TCAGGTGATGGGAATCATTG	240	243	169,881–170,120	15,410,2542–15,410,496
	R	TGTTCCAACCAAACAACCTG				
K20-1-1	F	CAGGTGTTCTACACTCCGAAC	89	92	170,032–170,120	15,410,405–15,410,496
	R	TGTTCCAACCAAACAACCTG				
K46-1	F	TGAGATAGCCGTCAAGATGCT	523	-	275,710–276,232	Absent
	R	AAGGACCACCATTCCATAGC

**Figure 2:**
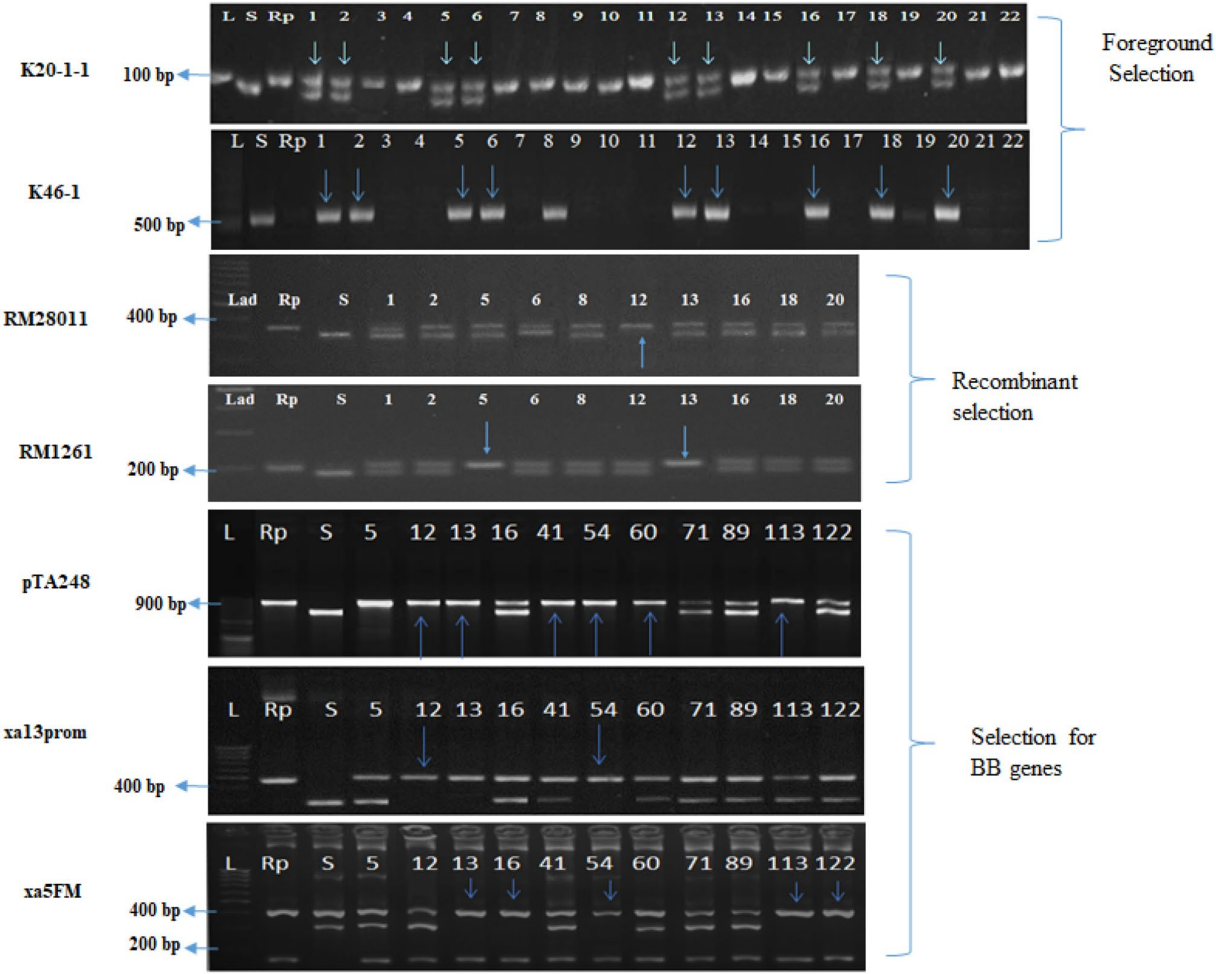
Molecular marker-based foreground, recombinant selections for *Pup1* QTL, and the bacterial blight resistance genes, *Xa21, xa13*, and *xa5* in BC_1_F_1_ generation. K20-1-1, a co-dominant marker linked to *Pup1* and K46-1, a functional marker of *Pup1* were used for foreground selection, RM18021 and RM1261 flanking the *Pup1* QTL on either side, on 12th chromosome were used for recombinant selection, pTA248, xa13prom, and xa5FM were used for the selection of positive plants for BLB resistance gene *Xa21, xa13*, and *xa5* respectively.

### Marker-assisted backcross breeding and foreground, recombinant, and background selection in backcross progenies

Hybridization was carried out between the donor parent Swarna and recipient parent ISM during *Rabi* (i.e. dry season) 2012–13 and detailed scheme of MABB followed is given in Fig. 1. A total of 29 F_1_s were genotyped with the co-dominant marker K20-1-1 and 20 plants were identified as true F_1_s (Table 2 and Fig. 2). The true F_1_s were then backcrossed with the recipient parent ISM and BC_1_F_1_s were produced. A total of 130 BC_1_F_1_ plants were subjected to foreground selection using the *Pup1* specific functional marker K46-1 and the co-dominant linked marker, K20-1-1 (Fig. 2), and 79 *Pup1* positive plants, which are in heterozygous condition (Table 2) were identified. They were then subjected to recombinant selection using the SSR markers flaking *Pup1* locus, viz., RM28011 and RM1261 (Fig. 2, Supplementary Fig. 3). A total of 11 plants (Table 2 and Supplementary Fig. 3) possessing a recombination event on one side of *Pup1* were identified (i.e. they were homozygous for ISM allele with respect to either RM28011 or RM1261). The foreground and recombinant positive BC_1_F_1_s were also screened for the presence of *xa5, xa13* and *Xa21* using xa5FM, xa13 prom, and pTA248 markers, respectively (Fig. 2). Two BC_1_F_1_ plants (viz., #A-13 and #A-54) were observed to be homozygous for all three bacterial blight (BB) genes (Table 2). The 11 plants were also analyzed for their background genome recovery using 66 parental polymorphic SSR markers (Table 2) and plant # A-13 was observed to possess the highest recurrent parent genome recovery of 78.03%. It was then utilized for backcrossing with ISM to generate BC_2_F_1_s.

**Table 2:** Details of F_1_ confirmation and foreground selection among the backcross derived plants for *Pup.*

Details of foreground selection (FS)													
Sl. No	Cross description		Generation			Total no. of seeds obtained		Total no. of seeds germinated and survived	Total no. positive plants (+ ve for *Pup1*)				
1	ISM*Swarna		F_1_			35		29	20				
2	ISM /( ISM*Swarna)		BC_1_F_1_			212		130	79				
3	ISM //( ISM*Swarna)		BC_2_F_1_			231		124	63				
4	ISM //( ISM*Swarna) selfed		BC_2_F_2_			392		231	76*				
Details of recombinant selection (RS)													
Sl. No.		Generation	Total number of plants screened			Number plants where recombination occurred at RM28011 end			Number plants where recombination occurred at RM1261 end^#^			Total number of plants Selected	
1		BC_1_F_1_	79			5			6			11	
2		BC_2_F_1_	63			4			-			4	
Details of Background selection (BS) and selection for BLB resistance in in BC_1_F_1_ generation											Details of percent background genome recovery (%RGP) in selected BC_2_F_2_plants		
Sl. No.	Foreground and Recombinant selection + ve plant #		No. of SSR makers screened	No. of SSR markers which are homozygous for ISM alleles	Background recovery (%)		Status for BLB resistance gene						
							*Xa21*		*xa13*	*xa5*			
1	A-5		66	33	75.00		*Xa21/Xa21*		*Xa13/xa13*	*Xa5/xa5*			
2	A-12		66	31	73.48		*Xa21/Xa21*		*xa13/xa13*	*Xa5/xa5*			
3	A-13		66	37	78.03		*Xa21/Xa21*		*xa13/xa13*	*xa5/xa5*			
4	A-16		66	31	73.48		*Xa21/xa21*		*Xa13/xa13*	*xa5/xa5*			
5	A-41		66	32	74.24		*Xa21/Xa21*		*Xa13/xa13*	*Xa5/xa5*	Sl. No.	BC_2_F_2_ plants	%RGP
6	A-54		66	34	75.76		*Xa21/Xa21*		*xa13/xa13*	*xa5/xa5*			
7	A-60		66	35	76.52		*Xa21/Xa21*		*Xa13/xa13*	*Xa5/xa5*	1	A-13-144-12	91.67
8	A-71		66	32	74.24		*Xa21/xa21*		*Xa13/xa13*	*Xa5/xa5*	2	A-13-144-53	95.45
9	A-89		66	35	77.27		*Xa21/xa21*		*Xa13/xa13*	*Xa5/xa5*	3	A-13-144-55	91.67
10	A-113		66	33	75.00		*Xa21/Xa21*		*Xa13/xa13*	*xa5/xa5*	4	A-13-144-82	93.18
11	A-122		66	32	74.24		*Xa21/xa21*		*Xa13/xa13*	*xa5/xa5*	5	A-13-144-112	93.18
Details of Background selection in BC_2_F_1_											6	A-13-144-135	94.70
Sl. No.	Foreground and Recombinant selection + ve plant			No. of SSR makers screened	No. of SSR markers which are homozygous for ISM alleles				Background recovery (%)		7	A-13-144-139	95.45
											8	A-13-144-170	94.70
1	A-13 -18			29	46				84.85		9	A-13-144-192	94.70
2	A-13-65			29	50				87.88		10	A-13-144-215	93.18
3	A-13-144			29	52				89.39		11	A-13-144-222	93.94
4	A-13-192			29	47				85.61		12	A-13-144-223	93.18

*In BC_2_F_2_ generation the number of plants homozygous for the *Pup1* locus, ^#^In BC_2_F_1_ no recombinant selection was carried for RM1216 because the plant selected in BC_1_F_1_ was already positive/recombine for RM126.

Crosses were made between ISM and Swarna in *Rabi* 2012–13, F_1_s obtained were confirmed for their hybridity using a co-dominant marker K 20-1-1. The true F_1_s were backcrossed to recurrent parent ISM to produce BC_1_F_1_. Obtained BC_1_F_1_s were subjected to FS and RS, the plants which were positive for FS and RS were genotyped for BLB and also subjected to BS. The best plant selected (A-13) was backcrossed to ISM, to produce BC_2_F_1_. The BC_2_F_1_s obtained was subjected to FS, RS, and BS, the best plant obtained (A-13-144) was selfeds to produce BC_2_F_2._

**Figure 3:**
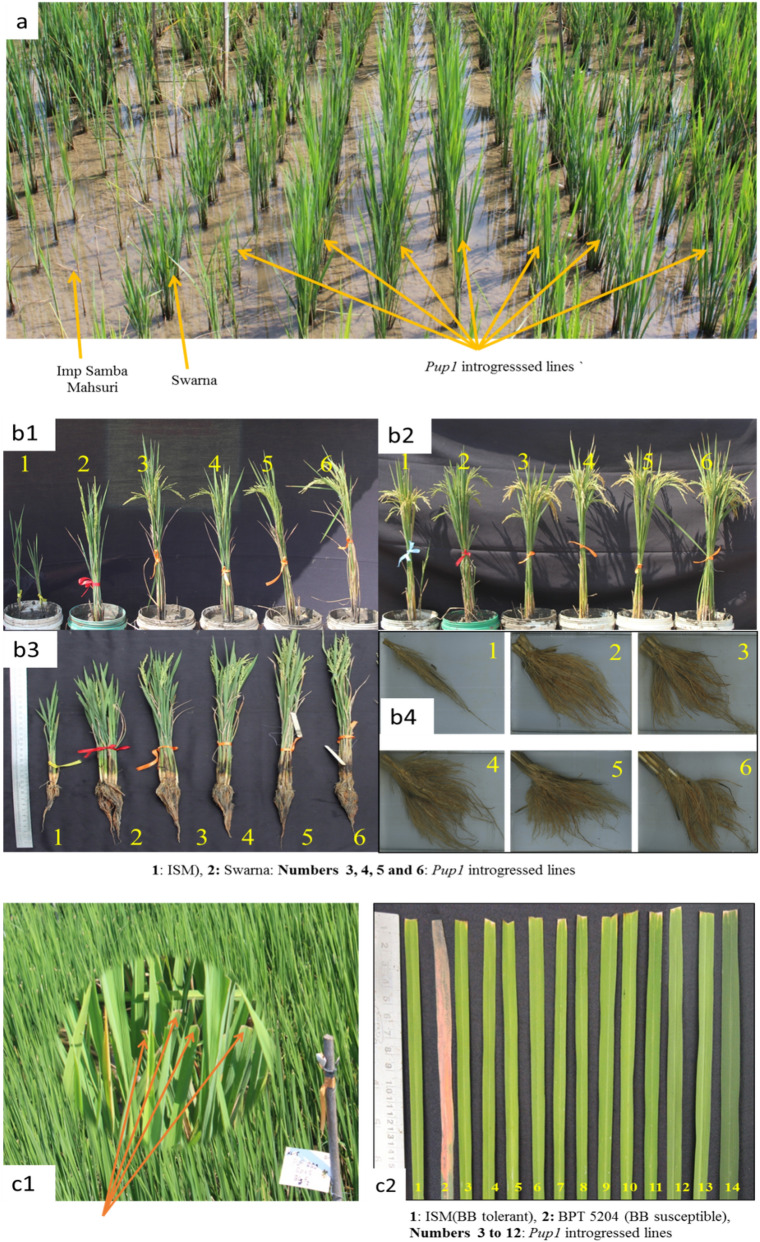
Performance of *Pup1* introgressed lines under low P (< 2 kg ha^−1^) and normal conditions (> 20 kg ha^−1^). **a:** ISM (Recipient/ Recurrent) showing poor growth and Swarna (Donor) and introgressed lines of ISM growing well under low P (< 2 kg ha^−1^) condition; **b1:** ISM, Swarna and introgressed lines under low P condition **b2:** ISM, Swarna and introgressed lines under normal P condition **b3:** Showing root and shoot length under low P condition; **b4:** Showing root volume under low P condition; **c1:** Screening for bacterial blight in the field, **c2**: Scoring of inoculated leaf.

A total of 124 BC_2_F_1_ plants were grown and subjected to foreground selection for *Pup1* using the marker K20-1-1 and a total of 63 plants were identified to be positive for the locus (Table 2)*.* Positive plants were further genotyped with the flanking marker, RM28011 (as the selected BC_1_F_1_ plant, i.e. # A-13 was earlier observed to be homozygous for ISM allele for the other flanking marker RM1261 in BC_1_F_1_ generation) in the recombinant selection. Four BC_2_F_1_ plants were observed to be homozygous for ISM specific allele with respect to the flanking marker, RM28011 (Table 2 and Supplementary Fig. 4). These plants (#A-13-18, #A-13-65, #A-13-144 and #A-13-192) were then analyzed for background genome recovery using the remaining 29 parental polymorphic SSR markers (which were still heterozygous for the ISM alleles in the selected BC_1_F_1_ plant # A-13; Table 2; Supplementary Fig. 4). The background recovery ranged from 84.85% to 89.23% among selected plants (Table 2) and a single plant #A-13-144 with maximum recurrent parent (ISM) genome recovery of 89.23% was selfed to generate BC_2_F_2_s. A total of 231 BC_2_F_2_ seedlings were analyzed with the co-dominant marker, K20-1-1, and a total of 76 plants that were homozygous for *Pup1* are identified (Table 2, Supplementary Fig. 5). Among them, twelve best plants (A-13-144-12, A-13-144-53, A-13-144-55, A-13-144-82, A-13-144-112, A-13-144-135, A-13-144-139, A-13-144-170, A-13-144-192, A-13-144-215, A-13-144-222, A-13-144-223), which were almost similar to the recurrent parent ISM, in terms of agro-morphological traits like plant height, days to fifty percent flowering, panicle length, number of tillers, grain yield, leaf color, grain type and grain hull color were advanced to BC_2_F_3_. The twelve BC_2_F_3_ lines were planted in both normal P and low P plots of ICAR-IIRR to evaluate their performance under both low as well as normal soil P and also to evaluate their BB resistance along with other agro-morphological features.

### Performance of *Pup1* introgressed lines under low soil P condition

The twelve best *Pup1 *introgressed BC_2_F_3_ ISM lines identified were evaluated under low P (< 2 kg ha^−1^) conditions along with the parents and checks. Under low P condition the traits viz., days to fifty percent flowering, number of productive tillers, flag leaf length, panicle length, root volume, dry root weight, dry shoot weight, thousand grain weight and grain yield per plant, showed significant difference among the introgressed lines with improved performance as compared to ISM. No differences were observed among the *Pup1* introgressed plants with respect to plant height, flag leaf width, root length and shoot length (Table 3). The introgressed lines were equivalent or superior to the original parent, ISM in respect of all the agro-morphological traits under low soil P condition (Table 3; Fig. 3).

**Table 3:** Evaluation of the *Pup1* positive homozygous BC_2_F_3_ lines in the low-P plot of ICAR-IIRR, Hyderabad.

Genotype/Entry	Days to fifty percent flowering (Days)	Plant height (cm)	Number of productive tillers (Nos.)	Flag leaf length (cm)	Flag leaf width (cm)	Panicle Length (cm)	Root length (cm)	Root volume (ml)	Dry root weight (g)	Dry shoot weight (g)	Shoot length (cm)	1000 grain weight (g)	Grain yield per plant (g)
A-13-144-12	125 ^C^	64.33 ^ABC^	6.67 ^D^	16.33 ^E^	1.2 ^AB^	17.33 ^E^	29.5 ^A^	31.12 ^AB^	2.61 ^ABCD^	21.92 ^A^	60.52 ^A^	12.53 ^FGH^	9.85 ^B^
A-13-144-53	120 ^EF^	63.67 ^ABC^	7.33 ^CD^	22.33 ^AB^	1.167 ^AB^	19.67 ^BC^	30.5 ^A^	28.62 ^BC^	2.78 ^AB^	21.63 ^AB^	60.12 ^A^	11.34 ^I^	9.95 ^B^
A-13-144-55	122 ^DE^	65.33 ^ABC^	7.33 ^CD^	21.33 ^BC^	1.2 ^AB^	19.33 ^BC^	33.25 ^A^	32.52 ^A^	2.91 ^A^	18.91 ^CD^	60.53 ^A^	14.76 ^D^	9.75 ^B^
A-13-144-82	125 ^C^	66.21 ^ABC^	7.33 ^CD^	19.67 ^CD^	1.1 ^B^	18.63 ^CDE^	32.61 ^A^	31.33 ^AB^	2.67 ^ABCD^	18.21 ^D^	63.22 ^A^	13.43 ^EF^	7.98 ^C^
A-13-144-112	128 ^B^	63.67 ^ABC^	7.55 ^CD^	18.33 ^DE^	1.17 ^AB^	18.43 ^CDE^	30.21 ^A^	30.14 ^AB^	2.23 ^E^	17.74 ^D^	60.21 ^A^	14 ^DE^	7.22 ^C^
A-13-144-135	125 ^C^	65.67 ^ABC^	7.33 ^CD^	18.61 ^DE^	1.2 ^AB^	18.67 ^CDE^	34.74 ^A^	26.31 ^C^	2.61 ^ABCD^	18.77 ^CD^	60.52 ^A^	12.49 ^FGHI^	9.65 ^B^
A-13-144-139	118 ^F^	70.67 ^AB^	11.33 ^A^	21.67 ^BC^	1.33 ^A^	21.51 ^A^	31.51 ^A^	31.2 ^AB^	2.49 ^BCDE^	20.52 ^ABC^	62.33 ^A^	13.54 ^EF^	12.55 ^A^
A-13-144-170	121 ^DE^	67.67 ^ABC^	8.22 ^BC^	20.25 ^BCD^	1.3 ^A^	20.67^AB^	32.57 ^A^	29.51 ^ABC^	2.49 ^BCDE^	19.45 ^BCD^	62.61 ^A^	11.76 ^GHI^	10.12 ^B^
A-13-144-192	122 ^DE^	67.67 ^ABC^	8.63 ^BC^	18.67 ^DE^	1.3 ^A^	20.33 ^AB^	33.54 ^A^	26.34 ^C^	2.39 ^CDE^	15.21 ^E^	60.41 ^A^	13.13 ^EFG^	10.58 ^B^
A-13-144-215	122 ^DE^	62.15 ^BCD^	7.51 ^CD^	20.33 ^BCD^	1.2 ^AB^	19.51 ^BC^	30.52 ^A^	29.36 ^ABC^	2.52 ^BCDE^	17.92 ^D^	59.53 ^A^	13.95 ^DE^	7.32 ^C^
A-13-144-222	125 ^C^	62.32 ^BCD^	7.5 ^CD^	18.5 ^DE^	1.21 ^AB^	17.5 ^DE^	30.62 ^A^	29.63 ^ABC^	2.49 ^BCDE^	17.33 ^DE^	58.84 ^A^	12.25 ^GHI^	7.58 ^C^
A-13-144-223	127 ^BC^	71.22 ^A^	7.5 ^CD^	24.71 ^A^	1.25 ^AB^	21.33 ^A^	31.32 ^A^	30.81 ^AB^	2.69 ^ABC^	21.24 ^AB^	63.5 ^A^	13.83 ^E^	8.42 ^C^
Imp Samba Mahsuri	127 ^BC^	41.29 ^F^	2.33 ^F^	12.32 ^G^	0.52 ^D^	11.31 ^H^	21.56 ^B^	12.57 ^DE^	0.96 ^G^	2.45 ^G^	37.02 ^D^	11.55 ^I^	2.59 ^E^
IR-64	116 ^G^	49.86 ^E^	3.41 ^E^	14.27 ^F^	0.85 ^C^	12.92 ^G^	20.28 ^B^	11.84 ^E^	0.47 ^H^	2.58 ^G^	44.58 ^C^	22.785 ^B^	2.95 ^DE^
MTU 1010	105.5 ^H^	55.57 ^D^	3.85 ^E^	17.08 ^E^	0.94 ^C^	14.95 ^F^	19.67 ^B^	14.27 ^D^	1.39 ^F^	4.53 ^F^	51.19 ^B^	23.88 ^A^	3.425 ^D^
Swarna	130 ^A^	63.60^C^	8.92 ^B^	18.34^DE^	1.22 ^AB^	18.67 ^CD^	30.49 ^A^	28.87 ^BC^	2.43 ^DE^	18.68 ^D^	61.14 ^A^	17.86 ^C^	9.93 ^B^
MSS Tests	8.79** (< .0001)	8.82 ^NS^ (0.4057)	1.43** (0.0014)	4.96** (0.001)	0.004 ^NS^ (0.3721)	1.88** (0.0006)	2.53 ^NS^ (0.761)	3.67* (0.039)	0.032* (0.036)	3.95** (0.0008)	2.25 ^NS^ (0.98)	2.29** (< .0001)	2.51** (< .0001)
MSS Checks/Controls	426.25**(< .0001)	353.51** (< .0001)	34.37** (< .0001)	29.57** (< .0001)	0.34** (< .0001)	40.38** (< .0001)	102.17** (< .0001)	259.5** (< .0001)	2.79** (< .0001)	243.70** (< .0001)	419.09** (< .0001)	126.69** (< .0001)	48.64** (< .0001)
MSS Tests vs Checks	168.59**(< .0001)	1211.9** (< .0001)	71.37** (< .0001)	142.28** (< .0001)	0.75** (< .0001)	167.8** (< .0001)	523.98** (< .0001)	1133.1** (< .0001)	10.85** (< .0001)	989.00** (< .0001)	1079.3** (< .0001)	215.52** (< .0001)	140.29** (< .0001)
Error	0.58	7.68	2.60	0.69	0.004	0.24	3.91	1.26	0.011	6.27	8.57	0.12	1.85
CV	0.63	4.75	7.74	4.78	5.85	2.93	7.40	5.01	5.62	5.92	5.44	2.05	5.90
CD tests @ 5%	2.35	8.54	1.43	2.57	0.19	1.50	-	3.45	0.32	2.27	-	1.05	1.21
CD Checks vs Tests @ 5%	2.35	8.54	1.43	2.57	0.19	1.50	6.10	3.45	0.32	2.27	9.02	1.05	1.21
Mean	122.40	62.56	7.06	18.92	1.14	18.17	29.56	26.53	2.26	16.07	57.89	14.58	8.11
Standard Error	1.46	1.93	0.55	0.76	0.05	0.72	1.18	1.74	0.17	1.66	1.84	0.94	0.72
Minimum	105.50	41.29	2.33	12.32	0.52	11.31	19.67	11.84	0.47	2.45	37.02	11.34	2.59
Maximum	130.00	71.22	11.33	24.71	1.33	21.51	34.74	32.52	2.91	21.92	63.50	23.88	12.55

**NS**: Non significant; **Highly significant; * Significant; Values under the parenthesis indicate the Probability value (Pr > F); Alphabets in the superscripts indicate LSD grouping.

The entry A-13-144-139 performed best under low P condition, followed by six equally performing genotypes viz., A-13-144-192, A-13-144-170, A-13-144-53, A-13-144-12, A-13-144-55 and A-13-144-135 with Swarna (Donor parent &Tolerant check).

### Performance of *Pup1* introgressed lines of ISM under normal soil P condition

Under normal soil P (> 25 kg P ha^−1^) level, *Pup1* introgressed BC_2_F_3_ lines were equivalent to the recurrent parent (ISM) in terms of days to fifty percent flowering, plant height, panicle length, shoot length and thousand-grain weight. The lines were observed to show equivalent or better performance as compared to ISM with respect to root volume, dry root weight, dry shoot weight, number of productive tillers, and grain yield (Supplementary Table 1, Fig. 3).

### Evaluation of *Pup1* introgressed lines of ISM lines for bacterial blight resistance

The *Pup1* introgressed BC_2_F_3_ lines along with bacterial blight resistant check (ISM) and bacterial blight susceptible check (Samba Mahsuri/ BPT 5204) were inoculated with a local virulent isolate of *Xoo,* (*Xanthomonas oryzae* pv*. oryzae*) DX-020 under field conditions (i.e. in soil with normal P). All the *Pup1* introgressed BC_2_F_3_ lines and the recurrent parent, ISM showed resistance reaction (lesion length of < 1 cm/IRRI-SES score of 1; Supplementary Table 2 and Fig. 3), while Samba Mahsuri showed susceptible reaction (Lesion length of 18.5 cm/IRRI-SES score of 9; Supplementary Table 2, Fig. 3).

### Evaluation of *Pup1* introgressed lines of ISM for grain and cooking quality

Four best BC_2_F_6_ *Pup1* introgressed ISM lines were evaluated for grain and cooking quality-related traits. The donor Swarna has medium bold type grains, while ISM is of medium-slender type grain. All the *Pup1* introgressed lines were observed to possess medium-slender grain and showed values equivalent to ISM in terms of all the grain and cooking quality parameters (Table 4, Supplementary Fig. 6).

**Table 4:** Grain quality parameters of the *Pup1* introgressed lines of ISM.

Grain quality component	Genotypes						Mean	SE
	A-13-144-53	A-13-144-170	A-13-144-139	A-13-144-192	Swarna	ISM		
Milling %	66.20	66.20	66.30	64.30	62.70	64.50	65.03	0.59
Head rice recovery	44.60	52.00	55.80	48.50	45.80	40.40	47.85	2.24
Kernel length	4.89	4.91	5.06	5.04	4.81	4.51	4.87	0.08
Kernel Breadth	1.68	1.80	1.88	1.81	2.11	1.68	1.83	0.07
L/B ratio	2.91	2.72	2.69	2.78	2.27	2.68	2.68	0.09
Volume expansion ratio (mm)	5.30	4.00	5.00	5.40	4.20	5.10	4.83	0.24
Water uptake (ml)	150.00	155.00	155.00	165.00	175.00	155.00	159.17	3.75
Kernel length after cooking (mm)	8.80	8.40	8.80	8.60	7.60	8.30	8.42	0.18
Elongation ratio	1.79	1.71	1.73	1.70	1.58	1.64	1.69	0.03
Alkali spreading value	4.00	4.00	4.10	4.00	4.00	4.00	4.02	0.02
Amylose content (%)	21.91	24.48	22.55	22.85	23.67	22.20	22.94	0.39
Gel consistency (mm)	22.00	22.00	22.00	22.00	30.00	22.00	23.33	1.33
Grain type	MS	MS	MS	MS	SB	MS	-	-
Grain chalkiness	A	A	A	A	VOC	A	-	-

MS and SB indicate medium slender and short bold respectively; A: Absent, VOC: Very occasionally present.

### Performance of a *Pup1* introgressed line of ISM in multi-location trials

One of the best performing lines of ISM with *Pup1* [viz., A-13-144-139 (designated as IET 28061)] was evaluated in multi-location trials of AICRIP in Kharif (i.e. wet season 2018) along with other entries and checks under both 100% and 50% of the recommended dose of phosphorus application at Cuttack (Odisha, India), Maruteru (Andhra Pradesh, India) and Hyderabad (Telangana, India). The line IET 28,061 performed remarkably well and recorded 16% and 30% yield advantage over the ISM under 100% and 50% dose of phosphorus application respectively. Further, the *Pup1* introgressed line performed equivalent to the donor parent, Swarna under both conditions (Table 5).

**Table 5:** Performance of *Pup1* introgressed ISM line under AICRIP trials at Odisha, Andhra Pradesh and Telangana under normal (100% RDF P application) and reduced (50% RDF P application).

Sl. No	Test Entries	Yield (in kg/ha)							
		Odisha (Cuttack)	Andhra Pradesh (Maruteru)	Telangana (Hyderabad)	Mean	Odisha# (Cuttack)	Andhra Pradesh (Maruteru)	Telangana (Hyderabad)	Mean
		At 100% RDF P application				At 50% RDF P application			
1	A-13-144-139 (IET 28061)^ѱ^	4780*	7920*	3728^$^	5476.00	4511*	8350*	2149^$^	5003.33
2	Swarna (Positive Check)	3026	8130	3026	4727.33	–	8005	2522	5263.50
3	ISM (Negative Check)	3733	6690	3662	4695.00	3767	5810	1952	3843.00
	Exp. Mean^β^	3619	6502	3995	4705.33	3227	6263	2184	3891.33
	CD	665.97	1604.86	375.03		452.31	1405.12	327.32	
	CV%	8.72	11.64	4.49		6.64	10.58	7.01	
	Percent improvement over ISM (recurrent parent)	28.05	18.39	1.80	16.63	19.75	43.72	10.09	30.19
	Percent improvement over Swarna	57.96	− 2.58	23.20	15.84	–	4.31	− 14.79	− 4.94

* Statistically Significant (*p* < 0.05) superior over the ISM; $ numerically superior over the ISM.

^#^ Data was not available for Swarna in Cuttack; the mean values presented in the table represent the mean of 23 entries which are involved in the AICRIP trial at that particular center; ^ѱ^ IET 28061 is the evaluation number given to A-13-144-139 derived ISM NIL of *Pup1* in AICRIP.

## Discussion

India has the largest area under cultivation of rice (~ 44 M ha) in the world. However, a significant proportion is either P-deficient or with a high P-fixing capacity^31^. The poor availability of P in the soil is one of the major yield-limiting factors in rice production^2^. The yield reduction due to low P can be overcome either by application of P fertilizers or by growing rice varieties which are P use efficient under low P availability. Application of more P fertilizer increases the production costs and associated import demands and also often results in fertilizer run-offs, which pollute the water bodies. Hence, the cultivation of P use efficient rice varieties could be the most economic and eco-friendly option for managing the problem. Rice is known to have significant variability for P uptake and use efficiency^11–13^. A major QTL, named *Pup1*, conferring tolerance to low soil P was identified^2^ and demonstration of better performance of *Pup1* containing rice lines^14^ indicates the scope for genetically improving the elite rice varieties, which are sensitive to low soil P.

ISM is a near-isogenic line of the very popular Indian rice mega-variety, Samba Mahsuri and ISM was earlier developed by our group through MABB by pyramiding three bacterial blight (BB) resistant genes, viz., *xa5*, *xa13* and *Xa21*. ISM retains the high yield potential and excellent grain quality attributes of Samba Mahsuri^19^ and it is steadily replacing Samba Mahsuri in the bacterial blight endemic areas in India and, presently occupying more than 130,000 hectares^32^. However, ISM was observed to be highly sensitive to low soil P in a study conducted at ICAR-IIRR, Hyderabad, India in the field plot with low soil P^33^. In order to improve the adoption of ISM in areas with low soil P and to increase the profit of farmers, in the present study, an attempt has been made to improve ISM for low soil P tolerance by targeted introgression of *Pup1* QTL through MABB approach.

The *Pup1* QTL has been identified as the major locus conferring low soil P tolerance and enhanced P uptake in rice^15^. Several markers have been developed for use in marker-assisted introgression of *Pup1* QTL into those rice varieties, lacking *Pup1*^14,18,34^. Most of the markers identified earlier for *Pup1*, including the functional markers, K46-1 and K46-2 were either dominant or CAPS based co-dominant markers, their deployment is difficult and cumbersome in routine MABB programs. The PCR-based marker K20-1 is located at a distance of 105 kb (i.e. < 1 cM) from the candidate gene for *Pup1* (i.e. the gene encoding a *PSTOL1*^17^). However, the amplicons derived from K20-1 needs to be subjected for restriction digestion with *Mse1* enzyme to distinguish tolerant and sensitive alleles in a co-dominant fashion^14^. In order to overcome the limitation of this marker, we amplified and sequenced the product derived from K20-1 among rice genotypes having *Pup1* and devoid of *Pup1*. It resulted in the identification of a unique 3-bp indel polymorphism nearer to reverse primer of K20-1 (Supplementary Fig. 1). Targeting this indel, a new marker, named K20-1-1 was developed and this marker showed a perfect co-segregation with the trait phenotype (Supplementary Fig. 2). The new marker, K20-1-1 is physically located quite close to the *Pup1* locus (~ 105 kb; < 1 cM) and was observed to clearly distinguish homozygous and heterozygous individuals with respect to *Pup1* as compared to the dominant functional marker, K46-1 (^14^; Fig. 2, Supplementary Fig. 2). Hence, we deployed this new marker in the process of marker-assisted introgression of *Pup1* into ISM.

Crosses were made between ISM and Swarna (the donor variety, possessing *Pup1*) and the F_1_s obtained were confirmed with the help of the co-dominant marker K20-1-1. Identification of true F_1_s using molecular markers is ideal as seeds that are set by accidental selfing which can be identified and removed quickly^35^. The true F_1_s confirmed through marker analysis were backcrossed to the recipient parent (ISM) to produce BC_1_F_1_s. Backcrossing is a method described by Harlan and Pope in 1922^36^ and it has been one of the widely used techniques in plant breeding for incorporating one or a few genes/traits into a well-adapted or elite crop variety^37,38^. The efficiency of selection in backcross breeding can be greatly increased by employment of molecular markers^39^, and in general, three levels of MABB have been described by Holland^40^ and Neeraja et al.^41^. The first level is ‘foreground selection’ where markers are used in combination with or to replace phenotypic screening for the target gene or QTL^42^. In the present study, screening for the target gene was done through initial screening with the *Pup1* linked marker K20-1-1. Later, the positive plants were reconfirmed with the *Pup1* specific, dominant, functional marker K46-1^14^. As phenotypic screening for low soil P tolerance in field conditions is laborious, hydroponic experiments are not much reliable^14^ and no defined criteria are available for screening for low soil P tolerance in segregating progenies, marker-assisted selection for *Pup1* is the preferred choice in gene introgression programs.

The second level of MABB involves the selection of desirable backcross plants possessing recombination on either one side or both sides of the target gene/QTL through a process called ‘recombinant selection’, with the help of markers flanking the target gene/QTL on either side at a genetic distance of ~ 5 cM^39^. Recombinant selection plays a crucial role in reducing the ‘linkage drag’, as in conventional breeding, the donor segment can tend to remain very large, even after many backcross generations, and hence the backcross plants may have many undesirable genes, which can negatively affect crop performance^43,44^. By using markers that flank the target gene, ‘linkage drag’ associated with the presence of the target gene/QTL can be minimized^39^. In the present study, two SSR markers RM28011 and RM1261, which are flanking the *Pup1* on either side (~ 1.5 Mb; equivalent to 5–6 cM) were used for the recombinant selection. In this study, we found only single recombinant events in BC_1_F_1_s, as the double recombinant events are rare and the sample size used for selection was also lesser than ideal^45^. In BC_2_F_1_, the recombinant selection was done with respect to the marker, RM28011 as the selected positive BC_1_F_1_ plant # A-13 already possessed recombination for the marker, RM1261. Thus, by the second backcross generation, plant(s) possessing recombination on either side of *Pup1* locus were identified, thus minimizing the linkage drag.

The third level of MABB involves ‘background selection’, in which backcross progenies with maximum recurrent parent genome recovery will be selected using markers that are unlinked to the target locus and spread across the entire genome^39^. This is useful as the recovery of the recurrent parent genome can be greatly accelerated and the number of backcrosses can be limited to just two or three^39^. In the present study, 66 rice SSR markers which are polymorphic between the donor and recurrent parents and distributed across the entire rice genome were used for background selection to accelerate the recovery of the recurrent parent genome and by BC_2_F_2_, plant(s) possessing a high level of recovery of ISM genome was obtained.

In addition to foreground selection for *Pup1,* marker-assisted selection was also carried out for the three BB resistant genes *xa5*, *xa13* and *Xa21* (which are present in ISM) in the first backcross generation. BB resistance is the essential character of the variety ISM and hence it is very crucial to retain this feature of the variety in the process of improving ISM for low P tolerance. The molecular markers, pTA248^24^, xa13 prom and xa5FM^25^ , which are also functional markers specific to BB resistance genes *Xa21*, *xa13,* and *xa5* respectively were employed to monitor the segregation of these genes among the backcross plants and by BC_1_F_1_, we were able to identify the plants, homozygous for the three bacterial blight resistance genes.

Twelve best BC_2_F_2_ plants, which were homozygous for *Pup1* and the three BB resistance genes and also possessing highest recovery of ISM genome were selfed to generate twelve BC_2_F_3_ lines and they were evaluated under low soil P (Available P < 2.0 kg ha^−1^) as well as under normal P (recommended dose of P application). A delay in flowering was observed in all the rice genotypes under low P condition in comparison to normal condition. The phenological delays have been reported for crop plants when exposed to low P condition^46–48^. It is predicted to be an adaptive mechanism of plants which leads to increased phosphorus acquisition and utilization^49^, helping crop plants to attain maximum reproductive biomass. Under the normal condition, all of the *Pup1* introgressed ISM lines recorded days to 50% flowering equivalent to ISM (Supplementary Table 1), while under low soil P condition, many introgression lines flowered earlier as compared to ISM (Table 3). This could be because of better uptake of P by *Pup1* introgressed ISM lines in the presence of *Pup1*^14,17^, increased phosphorus acquisition, and utilization of P, which could help to overcome the phenological delay noticed under low P condition.

A drastic reduction in the plant height, number of productive tillers, flag leaf width, flag leaf length, and panicle length was observed among the low soil P sensitive checks under low P condition in comparison to normal soil P condition. However, the low soil P tolerant check Swarna and the *Pup1* introgressed ISM lines did not show such a significant reduction in plant height (Table 3, Supplementary Table 1, and Fig. 3). A comparatively lesser reduction in the plant height, number of productive tillers, flag leaf width, flag leaf length, and panicle length was observed among the introgressed lines and Swarna. The height of the introgression lines was almost similar to ISM under normal condition, while under low P condition, plant height of *Pup1* introgressed ISM lines were significantly higher than their recurrent parent ISM. Availability of P significantly affects plant height^50^ and the presence of *Pup1* in the introgression lines helped them to acquire more P and maintain tissue growth in comparison to ISM under low P condition. The productive tillers and panicle length of *Pup1* introgressed ISM lines were significantly higher as compared to ISM under the low soil P condition. Once the plants sense the limited availability of P, they try to shorten their life cycle through early entry into the reproductive phase or delay the flowering to acquire the required threshold P to complete the life cycle. Some plants also change the metabolic machinery to utilize the available P efficiently to complete their life cycle, with few healthy seeds produced for perpetuation, thus resulting in lower tiller number and grain number. In the present study, the presence of *Pup1* in the introgression lines might have helped them in acquiring more and more P from the soil due to better foraging ability^14^, resulting in more productive tillers and panicle length and better yield under low P condition, as compared to ISM.

The root length of *Pup1* introgressed lines and tolerant check, Swarna was observed to be significantly better than ISM and other low P sensitive checks (MTU 1010 and IR-64) in the low P plot. Under normal soil P condition, significant differences were not noticed among the *Pup1* introgressed rice lines but they varied in comparison to checks analyzed. The root volume, dry root weight, shoot length and dry shoot weight of the *Pup1* introgressed lines was observed to be significantly higher as compared to the recipient parent, ISM and other low P sensitive checks (MTU 1010, IR-64) under low P condition (Table 3, Fig. 3). Interestingly, even in the normal soil P condition, the *Pup1* introgressed lines showed comparatively better performance for these traits as compared to ISM. Gamuyao et al.^17^ demonstrated that *Pup1* locus contains a major gene called Os*PSTOL1,* which greatly enhances the total root length and root surface area, which in turn helped the plants in acquiring more P under deficient condition. Acquisition of more P under low P condition helps the *Pup1* introgressed ISM plants to produce more dry matter as compared to ISM (which is devoid of *Pup1*). Further, it has also been reported that Os*PSTOL1* gene acts at least, partially independent of soil P levels, and is responsible for the increase of total root surface, even when the soil has sufficient levels of available P^17^. This may be one of the reasons for increased root volume and dry matter of *Pup1* introgressed lines as compared to ISM under normal conditions.

The grain weight (i.e. thousand-grain weight) of most introgressed lines was observed to be similar to ISM in normal condition and all the lines possessed medium slender grain type (Supplementary Fig. 6) with grain quality attributes similar to ISM. The grain yield of *Pup1* introgressed ISM lines was significantly higher as compared to ISM and other low soil P sensitive checks (MTU 1010 and IR-64) under low soil P condition. This improvement of yield under low P condition was mainly due to the presence of *Pup1* in the introgressed lines as reported earlier^14,51^. Under normal conditions, these lines performed equivalent to or better than the recipient parent ISM. This kind of improvement was also observed by Gamuyao et al*.*^17^, in the rice lines which are having Os*PSTOL1* as a transgene as compared to the original parent. Os*PSTOL1* is a *Pup1-*specific protein kinase gene, identified in the low soil P tolerant Indian rice variety Kasalath, and is not found in most of the low P sensitive modern varieties released for the irrigated ecosystem and also in Nipponbare, the sequenced reference japonica rice^17^.

Under low soil P condition, the *Pup1* introgressed line, A-13-144-139 recorded the highest yield, followed by, A-13-144-192, A-13-144-170, A-13-144-53, A-13-144-12, A-13-144-55, and A-13-144-135 and there was no significant difference among these six lines for grain yield. Similarly, under normal P application, the highest grain yield was recorded in the introgression line, A-13-144-139 followed by A-13-144-170, A-13-144-112, A-13-144-53, A-13-144-135, A-13-144-55, and A-13-144-192; however, the yield differences were not significant. The lines which were performing well under both normal and low soil P condition and possessing agro-morphological characters similar to ISM viz., A-13-144-53, A-13-144-139, A-13-144-170, and A-13-144-192 were then advanced further for critical evaluation.

All the *Pup1* introgressed BC_2_F_3_ lines and the recurrent parent, ISM showed resistance reaction (lesion length of < 1 cm with IRRI-SES score of 1) against DX-020 (Supplementary Table 2 and Fig. 3), while Samba Mahsuri (BPT 5204) showed susceptible reaction (lesion length of 18.5 cm with an IRRI-SES score of 9). ISM is an EDV of Samba Mahsuri, bred to achieve resistance against BB in the genetic background of Samba Mahsuri, through pyramiding of three major and effective BB resistance genes viz., *Xa21*, *xa5*, *xa13* through MABB^19^. In our study, molecular markers specific to these genes were gainfully used to retain the three major BB resistance genes in the *Pup1* introgressed lines of ISM. Grain and cooking quality analysis of the introgressed lines of ISM revealed that they are almost similar in quality aspects in comparison to ISM (Table 4, Supplementary Fig. 6). All four selected backcross derived lines of ISM had the highly desirable medium-slender grain type, absence of chalkiness, and gel consistency similar to ISM.

From the selected advanced backcross derived lines of ISM possessing *Pup1,* one superior line, A-13-144-139 (IET 28061) was evaluated in multi-location trials of AICRIP in the year 2018 (in wet season 2018) for its performance over the locations. IET 28061 preformed significantly better under both normal soil P condition as well as under a reduced dose of P application. Based on the excellent performance, it has been promoted to the third year of testing (i.e. final year of testing) in the wet season 2019). After validation under low soil P and normal soil P conditions for another year, the entry, IET 28061 will be recommended and identified for release as a variety for commercial cultivation.

The backcross derived lines of ISM, possessing *Pup1* developed through this study are also being used as genetic stocks in the improvement of other elite rice lines for low P tolerance and BB resistance, as these lines can be used for simultaneous improvement of both bacterial blight resistance and low soil P tolerance in marker-assisted selection programs. Earlier, through another similar study, we demonstrated that backcross derived lines possessing *Pup1* in the genetic background of another Indian mega-variety of rice, MTU1010 show higher and better root-associated traits^51^. Such breeding lines of ISM and MTU1010 possessing *Pup1* and performing well in soils with low available P will enhance the yield in the areas where available soil P is low to moderate. Moreover, reduced application of P will help in cutting down the application of P fertilizers significantly, thus helping in a reduction in the cost of production and reduction in the environmental pollution due to P fertilizer run-offs. The two studies also demonstrated that MABB can be effectively utilized in improving elite varieties with greater precision, by introgressing the traits lacking in them to make them better yielding and resistant/tolerant to multiple stresses and ensure that they are more beneficial to the farmers.

## Conclusion

As P resources are recognized as one of the limiting resources in the near future, the development and use of low soil P tolerant varieties of crop plants are gaining greater importance. Varietal improvement for tolerance to low soil P in major staple crops like rice helps in cutting down the production costs, import burdens, and environmental contaminations due to fertilizer run-offs. In the present study, MABB approach was successfully utilized in improving low soil P tolerance of an Indian elite rice variety ISM, while retaining its yield, grain, and cooking quality and BB resistance. The low soil P tolerant ISM line performing well in P deficient condition in multi-location trials ensures the possibility of growing rice under reduced application of P fertilizers, P deficient soils, and problematic soils for getting sustainable yields, thus enhancing the income of farmers.

## Material and methods

### Plant materials

Improved Samba Mahsuri (ISM), a high-yielding, bacterial blight resistant, fine-grain type, low glycemic rice variety, developed and released by ICAR-Indian Institute of Rice Research (ICAR-IIRR), Hyderabad, India, and CSIR-Centre for Cellular and Molecular Biology (CSIR-CCMB), Hyderabad, India^19^ was used as the recurrent parent. ISM, being a near-isogenic line of the very popular, Indian rice mega-variety, Samba Mahsuri is steadily replacing the original rice variety in bacterial blight endemic areas. However, it is highly sensitive to low soil P and hence its adoption is limited in soils prone to low availability of P. Swarna (MTU 7029), a high yielding, mega rice variety of India, which has good tolerance to low soil P and possessing *Pup1* (major QTL associated with low soil P tolerance^20^, was used as the donor parent in the marker-assisted backcross breeding program.

### Development of new co-dominant marker for *Pup1*, K20-1-1

The PCR products derived from the CAPS marker K20-1^14^ from Vandana (low soil P tolerant genotype, possessing *Pup1*) and ISM (Low soil P sensitive genotype, which is devoid of *Pup1*) were subjected for Sanger’s sequencing. Initially, the desired fragments of interest were eluted from 1% TAE agarose gel, purified using PCR Clean-Up System (Promega) and cloned into pGEM-T easy vector (Promega) and sequenced using an automated DNA sequencer (Perkin Elmer, MA; Bioserve Biotechnologies, India). A multiple sequence alignment, of the sequences from Vandana and ISM with reference genome of Nipponbare and Kasalath corresponding to *Pup1* region, retrieved from NCBS database, was carried out using CLUSTALW^21^ and physical positions of the amplicons were derived using BioEdit version 7.9^22^. The primer3 version 0.4.0 software^23^ was used to design the new primer pairs by targeting an indel region.

### Marker-assisted backcross breeding for targeted transfer of *Pup1* into Improved Samba Mahsuri

A cross was made between recurrent parent ISM and donor parent Swarna and the true F_1_s were identified with the help of the co-dominant marker, K20-1-1. The true F_1_s were backcrossed with the recurrent parent ISM to develop BC_1_F_1_s. Foreground selection for *Pup1* in BC_1_F_1_ was carried out using co-dominant marker K20-1-1 and the positive plants were reconfirmed with the dominant, functional marker K46-1^14^. The positive BC_1_F_1_ plants, were then analyzed for bacterial blight resistance using the functional markers specific for bacterial blight resistance genes *Xa21* (pTA248^24^), *xa13* (xa13prom^25^ and *xa5* (xa5FM^25^). Such plants were subjected to recombinant selection using the SSR markers RM28011 and RM1261, flanking the *Pup1* locus on either side (~ 1.5 Mb) to identify the recombinants with minimum linkage drag. The foreground and recombinant selected plants were then subjected to background selection using parental polymorphic SSR markers (n = 66) which were distributed across the rice genome to identify those positive plants, which have maximum recovery of the recurrent parent genome as described in Sundaram et al.^19^. The process of MABB involving foreground, recombinant, and background selections, was repeated to produce BC_2_F_1_s, wherein a single plant possessing *Pup1*, the bacterial blight resistance genes and recombination event on both sides of *Pup1* and also possessing the maximum recovery of ISM genome were identified and selfed to get BC_2_F_2_s. They were then analyzed with the co-dominant marker K20-1-1 to identify plants homozygous for *Pup1*. The plants homozygous for *Pup1* were selfed to produce BC_2_F_3_s. The BC_2_F_3_ plants were then screened for low P tolerance, bacterial blight resistance, key agro-morphological traits, and grain type and the superior lines were advanced through the pedigree method of selection. A set of superior BC_2_F_6_ lines were analyzed for grain and cooking quality as explained in Sundaram et al.^19^. The Miniprep protocol recommended by Zheng et al.^26^ was followed for DNA isolation from the parents, F_1_s, and backcross-derived plants. PCR protocol described in Chin et al.^14^ was adopted for the marker K46-1, while for the amplification of rice SSR markers, the protocol described in Sundaram et al.^19^, was followed. The PCR protocol of initial denaturation 94 °C for 2 min, followed by 35 cycles of denaturation at 94 °C for 30 s, annealing at 55 °C for 30 s and extension at 72 °C for 45 s with a final extension at 72 °C for 5 min was adopted for K20-1-1. The amplified product of K46-1 and SSR markers were resolved in 1.2% and 3.5% Seakem LE agarose gel (Lonza, USA), respectively. The product of K20-1-1 was resolved in native PAGE (8%) for better resolution.

### Evaluation of *Pup1* introgressed ISM lines for Low P tolerance

The seeds of selected BC_2_F_3_ lines were sown in nursery and 30 days old seedlings were transplanted into low soil P plot (Available P < 2 kg ha^−1^, tested before planting) as well as in normal soil P plot (Available P > 20 kg ha^−1^) in the experimental research farm at ICAR-Indian Institute of Rice Research (ICAR-IIRR) Hyderabad, India, in an augmented design with four checks, viz., Swarna, ISM, MTU 1010 and IR64. Standard agronomic practices were followed to raise a healthy crop in both normal and low soil P plots, except that no P fertilizer was applied to the low soil P plot. Pre-harvest agro-morphological character like days to 50% flowering (DFF), plant height (PH), number of tillers per plant (NT), flag leaf length (FLL), flag leaf width (FLW), panicle length (PL), shoot length (SL), root length(RL) and root volume (RV), dry shoot weight and dry root weight were recorded during the crop growth. After harvesting the plants, yield per plant, grain type, and 1000-seed weight were recorded. The average root length was measured by carefully uprooting the plants without causing any damage to the root system, washing in running water and measuring the length from the crown of the root to the root tip. The root volume was measured by using the water displacement method, wherein roots were dried in shade and then in a hot air oven and weighed to record the root dry weight. The data were analyzed using SAS 9.2 (SAS version 9.2 software packages, SAS Institute, Inc.; Cary, NC).

### Evaluation of *Pup1* introgressed ISM lines for bacterial blight resistance

The backcross derived plants grown in normal soil P plot were inoculated with a virulent isolate of the bacterial blight pathogen, *Xanthomonas oryzae* pv. *oryzae* (*Xoo*) DX-020. The *Xoo* strain was cultured and stored as described by Laha et al.^27^. The rice plants were clip inoculated with a bacterial suspension of 10^8–9^ cfu/ml at the maximum tillering stage as described in Kauffman et al.^28^. Approximately 5–10 leaves were inoculated per plant, and disease reaction was scored after 14 days from the inoculation. The disease score was also calculated as per the IRRI standard evaluation system (IRRI-SES), based on the percent diseased leaf area^29^.

### Evaluation of a selected *Pup1* containing line of ISM in multi-location trials

A selected NIL of ISM possessing *Pup1,* namely, A-13-144-139, which had the grain and cooking quality, agro-morphological traits and yield equivalent to ISM, was nominated for AICRIP trials. In this trial, a total of 21 entries derived from different ongoing rice breeding programs all over India were tested for their performance under normal (100% recommended dose of P) and reduced application of P (50% of the recommended dose of P), along with controls^30^. ISM was used as a negative control and Swarna was used as a positive control in the experiments.

## Supplementary information

Supplementary Information.
