# Supplementary material for: Marker assisted improvement of low soil phosphorus tolerance in the bacterial blight resistant, fine-grain type rice variety, Improved Samba Mahsuri
Source: Sci Rep. 2020 Dec 3;10:21143. doi: 10.1038/s41598-020-78186-5 (PMC7713241; doi:10.1038/s41598-020-78186-5)
Supplement: Supplementary file 1 — Supplementary Information. [file 41598_2020_78186_MOESM1_ESM.pdf]

Title: Marker assisted improvement of low soil phosphorus tolerance in the bacterial blight resistant, fine-grain type rice variety, Improved Samba Mahsuri

Mahadeva Swamy H.K.[1&3*], Anila M.[1*], Kale R.R.[1*], Rekha[1] G, Bhadana V.P.[1 & 2*], Anantha M.S.[1], Brajendra P.[1], Balachiranjeevi C.H.[1], Hajira S.K.[1], Laxmi Prasanna B.[4], Pranathi K.[1], Dilip T.[1], Kousik M.B.V.N.[1], Harika G.[1], Surekha K.[1], Mahender Kumar R.[1], Cheralu C.[4], Gouri Shankar V.[4], Laha G.S.[1], Prasad M.S.[1], Subba Rao L.V.[1], Madhav M.S.[1], Balachandran S. M.[1], Sundaram R.M.[1*#]

*Authors contributed equally.

1. ICAR-Indian Institute of Rice Research (IIRR), Rajendranagar, Hyderabad 500030, India.
2. ICAR-Indian Institute of Agricultural Biotechnology (IIAB), Ranchi-834010, India.
3. ICAR- Sugarcane Breeding Institute (SBI), Coimbatore-641007, India.
4. College of Agriculture, PJTSAU, Rajendranagar, Hyderabad 500030, India

[#] **Corresponding author:**

Dr. R. M. Sundaram, Principal Scientist, Crop improvement section, ICAR-IIRR, Hyderabad, Pincode -500030, India. Email: rms_28@rediffmail.com Tel (O): 91 40 24591226 Fax (O): 91 40 24591217

Supplementary Figure 1 : Alignment of sequences of amplicons of K20-1 from ISM and Vandana, with Japonica and Kasalath sequences corresponds *Pup1* locus on chromosome 12

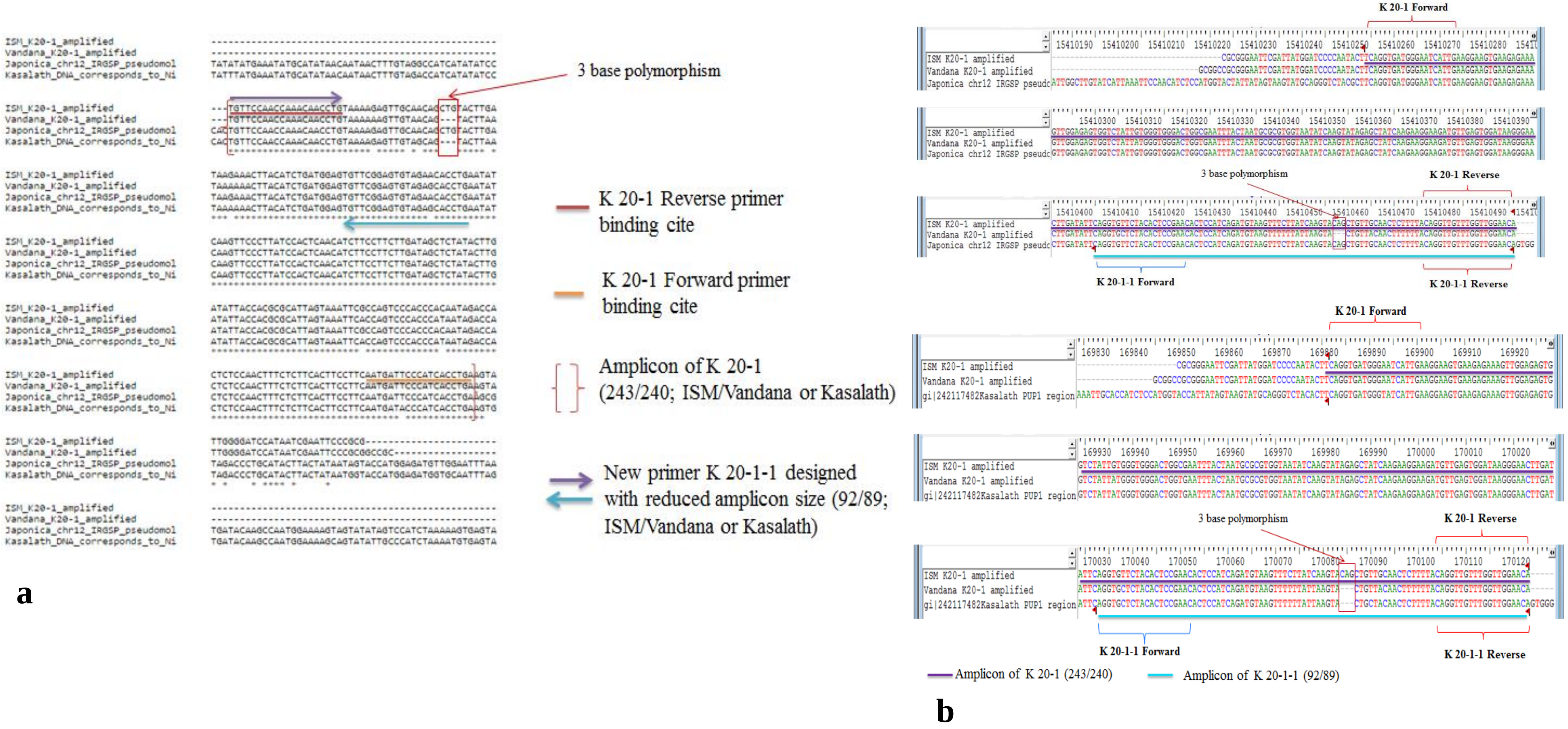

a: ClustalW alignment of sequence derived from K20-1 amplified fragment of ISM and Kasalath with Japonica sequence from IRGSP pseudomolecule of 12th Chromo and Kasalath sequence corresponds to *Pup1* region (GI: 42117482/AB458444.1). Primer binding regions and 3 base pair deletion regions from ISM and Vandana showed perfect alignment with corresponding reference sequence from Kasalath and Japonica. The red and orange line indicates the primers/primer binding sites of K 20-1; Blue and purple arrows indicates new primers/ new primer binding sites. The original K20-1 amplifies the fragment of 243/240, whereas the new primers designed (K 20-1-1) amplifies an amplicon of 92/89 which can easily resolve three base pair polymorphism.

b: Physical positions of new and old primer sequence with respect reference sequence Japonica and reference sequence Kasalath (GI: 42117482/AB458444.1) on 12th chromosome corresponds to *Pup1* region on 12th chromosome

Supplementary figure 2: Amplification patterns of K46-1 and K20-1-1 in F_2 population of ISM/Swarna

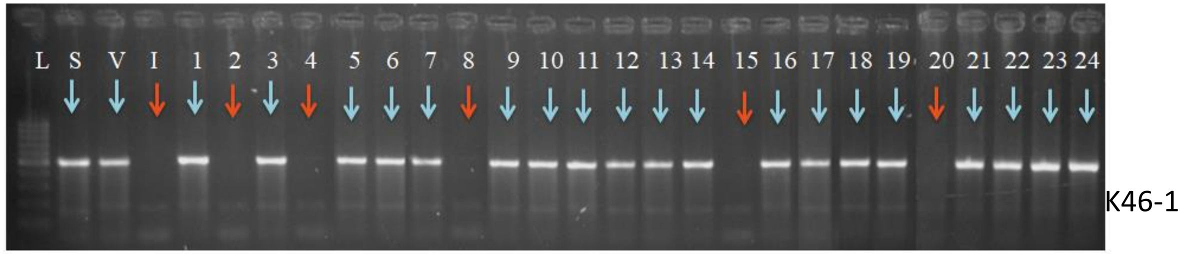

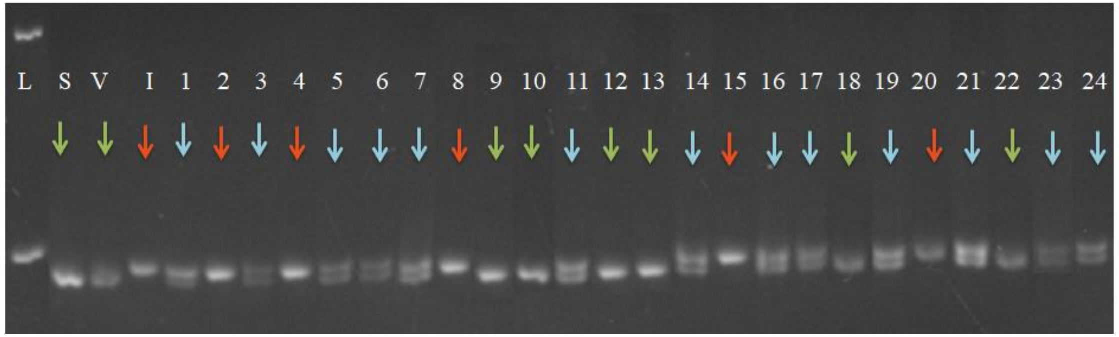

L – 100 bp ladder
S – Swarna (Donor)
V – Vandana (Another Donor for *Pup1*)
I –Improved Samba Mahsuri (Recipent)
1 to 28 – BC_2F_2 plants of ISM and Swarna
Blue arrow – Presence of *Pup1*
Red arrow – Absence of *Pup1*
Green arrow – Presence of *Pup1* in homozygous condition

Supplementary Figure 3: Graphical representation of Recombinant and Background selection on BC_1F_1 generation

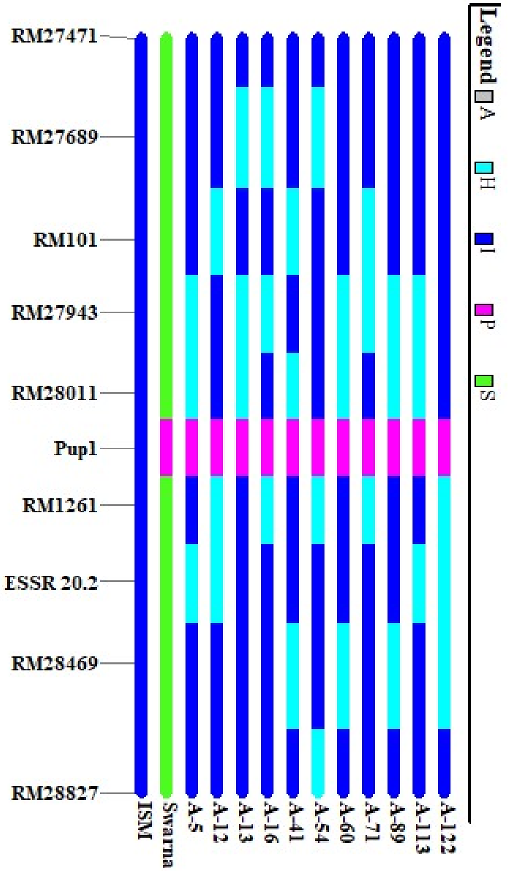

H: Heterozygote

I: Imp Samba Mahsuri specific allele

P: *Pup1*QTL

S: Swarna specific allele

The BC_1F_1 plants A-5, A-13, A41, A60, A-89 and A-113 had a recombination event at RM1261 side of *Pup1* and A-12, A-16, A-54, A-71 and A-122 had recombination event at RM28011 side of *Pup1*. The line A-13 which had recombination event at one end (RM1261) and had maximum genome recovery (78.03 %) of recurrent parent ISM genome was selected and backcrossed to produce BC_2F_1s

Supplementary Figure 4: Graphical representation of Recombinant and Background selection on BC_2F_1

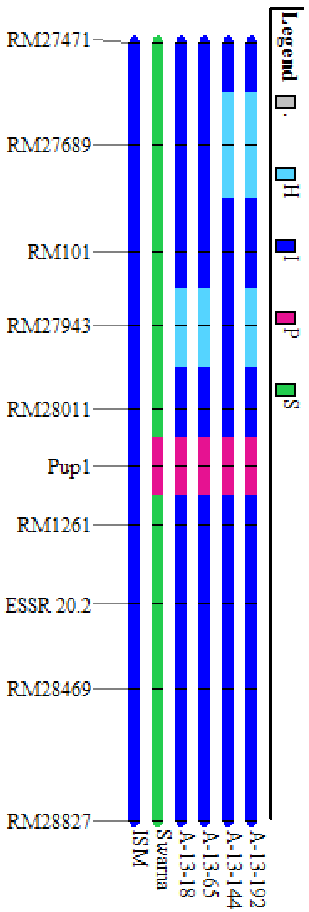

H: Heterozygote

I: Imp Samba Mahsuri specific allele

P: *Pup1* QTL

S: Swarna specific allele

The BC_1F_1 plants A-13-18, A-13-65, A-13-144 and A-13-192 had recombination event at both RM1261 side and RM28011 ends of *Pup1*. The line A-13-144 had recombination event at both ends of *Pup1* and maximum genome recovery (89.39 %) of recurrent parent ISM genome was selected and selfed to produce BC_2F_2s.

Supplementary Figure 5: Selection of plants possessing *Pup1* in homozygous condition in BC_2F_2 plants using *Pup1* specific co-dominant marker K20-1-1

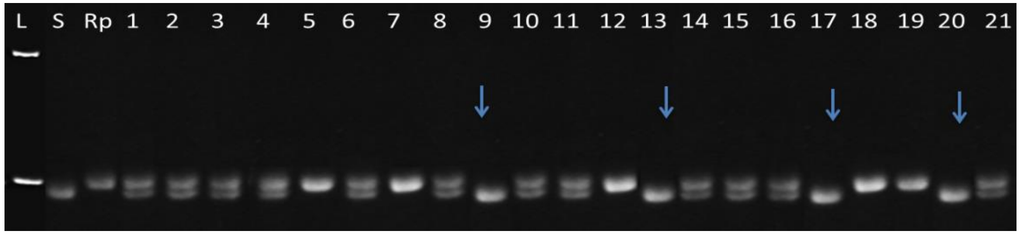

L – 100 bp ladder
S – Swarna (Donor)
Rp – ISM (Recipient)
Numbers – BC_2F_2 plants of ISM and Swarna
Blue arrow indicates BC_2F_2 plants possessing *Pup1* in homozygous condition

Supplementary figure 6: Grains and kernel characteristics of recipient, donor and *Pup1* introgressed BC_2F_6 ISM lines grown under normal plot

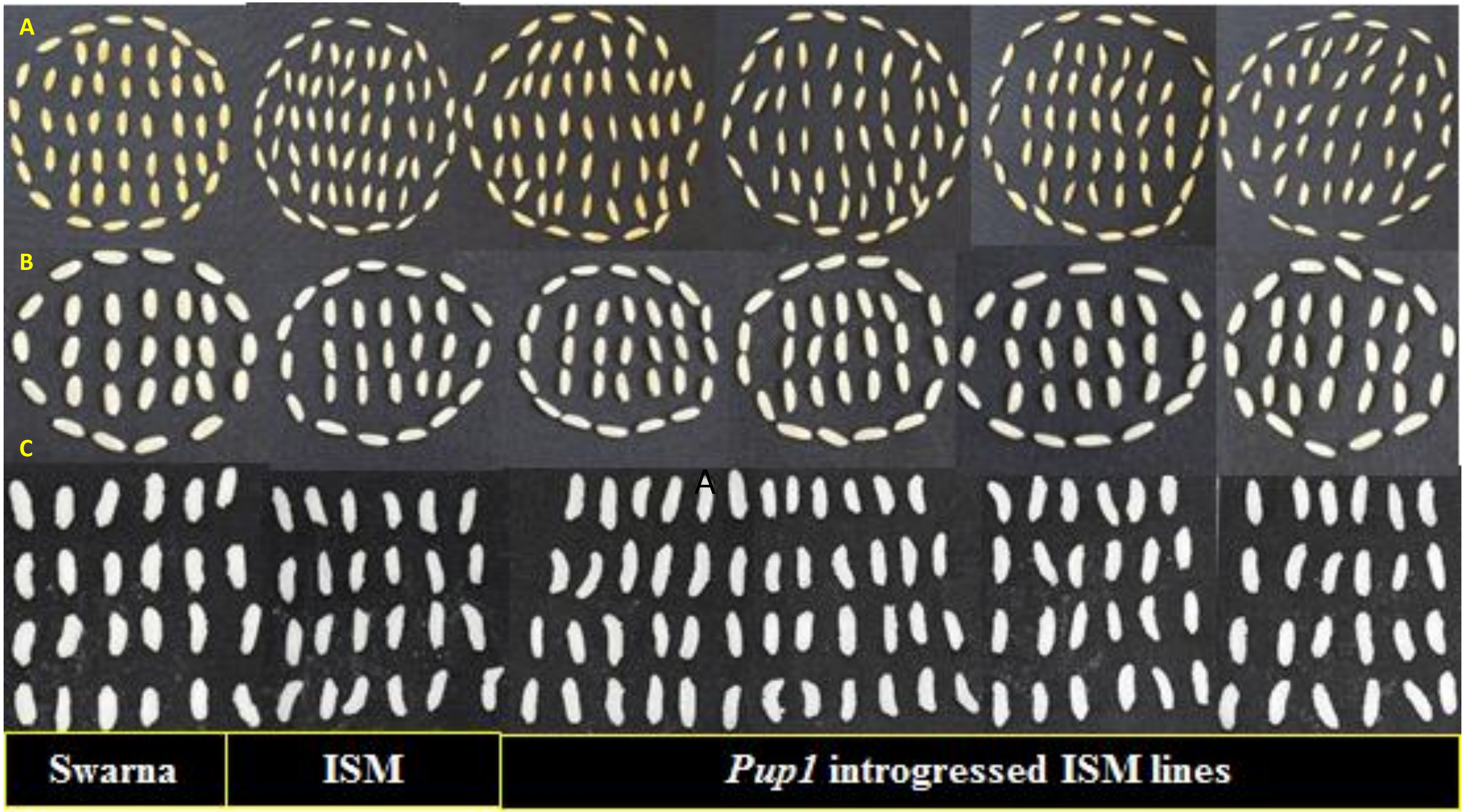

A: Whole grain; B: De-husked Kernels; C: Cooked Rice

Swarna (Donor) with medium bold grain type, Improved Samba Mahsuri (ISM) and Pup1 introgressed Imp Samba Mahsuri lines with medium slender grain type

Supplementary table 1: Evaluation of the *Pup1* positive homozygous BC_2F_3 lines in normal plot of ICAR-IIRR

Genotype/Entry	Days to fifty percent flowering (Days)	Plant height (cm)	Number of productive tillers (Nos.)	Flag leaf length (cm)	Flag leaf width (cm)	Panicle Length (cm)	Root length (cm)	Root volume (ml)	Dry root weight (g)	Dry shoot weight (g)	Shoot length (cm)	Thousand grain weight (g)	Grain yield per plant (g)
**A-13-144-12**	115.00 ^{B}	67.67 ^{E}	13.33 ^{ABC}	23.33 ^{BCD}	1.27	22.00 ^{ABC}	25.6	91.34 ^{A}	7.51 ^{A}	36.67 ^{BCD}	65.53 ^{EF}	12.44 ^{H}	26.17 ^{BCDEF}
**A-13-144-53**	113.00 ^{BC}	77.67 ^{BCD}	11.00 ^{CDEF}	26.21 ^{AB}	1.5	21.67 ^{ABC}	26.6	90.61 ^{A}	7.61 ^{A}	35.12 ^{CD}	72.34 ^{BCD}	13.85 ^{DEFGH}	27.16 ^{ABC}
**A-13-144-55**	115.00 ^{B}	74.00 ^{CDE}	11.33 ^{CDEF}	21.32 ^{CD}	1.2	21.33 ^{BC}	27.3	93.45 ^{A}	8.12 ^{A}	39.41 ^{ABC}	67.81 ^{DEF}	14.96 ^{D}	26.91 ^{ABCD}
**A-13-144-82**	106.00 ^{D}	83.33 ^{B}	12.33 ^{ABC}	24.11 ^{BCD}	1.2	21.67 ^{ABC}	26.4	93.80 ^{A}	8.12 ^{A}	40.66 ^{ABC}	77.10 ^{AB}	13.01 ^{FGH}	21.62 ^{GHI}
**A-13-144-112**	113.00 ^{BC}	83.00 ^{B}	14.00 AB	20.33 ^{D}	1.7	20.67 ^{BC}	25.4	90.41 ^{A}	7.43 ^{A}	41.84 ^{AB}	77.30 ^{AB}	14.12 ^{DEFG}	28.34 ^{ABC}
**A-13-144-135**	112.00 ^{BCD}	82.67 ^{B}	11.56 ^{BCDE}	23.52 ^{BCD}	1.3	21.25 ^{BC}	26.8	88.17 ^{A}	7.34 ^{A}	40.49 ^{ABC}	76.85 ^{AB}	14.70 ^{DE}	26.99 ^{ABCD}
**A-13-144-139**	115.00 ^{B}	79.67 ^{BC}	14.12 ^{A}	28.21 ^{A}	1.5	21.56 ^{ABC}	25.4	93.24 ^{A}	8.07 ^{A}	42.63 ^{A}	72.12 ^{BCD}	14.21 ^{DEFG}	30.13 ^{A}
**A-13-144-170**	112.00 ^{BCD}	76.00 ^{BCDE}	11.67 ^{ABCD}	24.19 ^{ABCD}	1.39	20.67 ^{BC}	26.5	92.13 ^{A}	7.38 ^{A}	36.46 ^{BCD}	71.33 ^{CD}	14.52 ^{DEF}	28.75 ^{AB}
**A-13-144-192**	112.00 ^{BCD}	77.33 ^{BCD}	11.00 ^{CDEF}	25.21 ^{ABC}	1.35	21.33 ^{BC}	27.23	88.59 ^{A}	7.47 ^{A}	38.14 ^{ABC}	70.60 ^{CDE}	14.47 ^{DEF}	26.69 ^{ABCDE}
**A-13-144-215**	114.00 ^{B}	79.00 ^{BC}	13.00 ^{ABC}	26.65 ^{AB}	1.3	22.33 ^{ABC}	26.12	93.20 ^{A}	8.07 ^{A}	40.25 ^{ABC}	72.33 ^{BCD}	12.84 ^{GH}	22.96 ^{EFGI}
**A-13-144-222**	112.00 ^{BCD}	82.33 ^{BC}	12.00 ^{ABC}	25.21 ^{ABC}	1.34	21.33 ^{BC}	25.53	91.85 ^{A}	7.45 ^{A}	38.49 ^{ABC}	75.18 ^{ABC}	13.33 ^{EFGH}	24.99 ^{BCDEFG}
**A-13-144-223**	112.00 ^{BCD}	70.33 ^{DE}	12.33 ^{ABC}	21.23 ^{CD}	1.28	20.44 ^{BC}	26.12	93.21 ^{A}	8.07 ^{A}	37.19 ^{ABCD}	65.34 ^{F}	14.38 ^{DEFG}	24.64 ^{CDEFG}
**Imp Samba Mahsuri**	116.25 ^{B}	78.18 ^{BC}	9.91 ^{DEF}	24.11 ^{BC}	1.46	19.90 ^{C}	23.62	42.84 ^{D}	3.45 ^{D}	27.11 ^{E}	71.39 ^{CD}	14.38 ^{DE}	20.66 ^{I}
**IR-64**	108.25 ^{CD}	76.94 ^{BCD}	9.65 ^{EF}	22.77 ^{CD}	1.23	22.96 ^{AB}	24.82	43.13 ^{D}	3.55 ^{D}	28.43 ^{E}	70.51 ^{D}	23.04 ^{B}	22.05 ^{GI}
**MTU 1010**	98.25 ^{E}	90.94 ^{A}	9.40 ^{F}	23.67 ^{BC}	1.24	24.20 ^{A}	26.14	54.95 ^{C}	4.53 ^{C}	33.18 ^{D}	77.58 ^{A}	24.60 ^{A}	24.05 ^{DEFH}
**Swarna**	125.00 ^{A}	77.17 ^{BC}	9.78 ^{DEF}	25.08 ^{AB}	1.36	24.20 ^{A}	29.07	68.50 ^{B}	5.56 ^{B}	37.45 ^{BC}	69.50 ^{DE}	20.15 ^{C}	23.44 ^{FG}
**MSS Tests**	5.90 ^{NS} (0.30)	25.74 * (0.02)	1.19 ^{NS} (0.16)	5.58 * (0.03)	0.02 ^{NS} (0.20)	0.31 ^{NS} (0.99)	0.45 ^{NS} (0.99)	3.61 ^{NS} (0.77)	0.11 ^{NS} (0.57)	5.37 ^{NS} (0.22)	17.65 * (0.002)	0.66 ^{NS} (0.08)	5.83 * (0.02)
**MSS Checks/Controls**	520.23 ** (<.0001)	183.78 ** (<.0001)	0.19 ^{NS} (0.83)	3.67 ^{NS} (0.15)	0.08 ** (0.009)	16.49 ** (0.0006)	21.99 * (0.002)	590.83 ** (<.0001)	3.89 ** (<.0001)	89.30 ** (<.0001)	53.00 ** (<.0001)	81.16 ** (<.0001)	9.17 * (0.01)
**MSS Tests vs Checks**	2.86 ^{NS} (0.43)	64.10 * (0.01)	47.15 ** (<.0001)	0.33 ^{NS} (0.67)	0.03 ^{NS} (0.18)	14.62 ** (0.006)	0.77 ^{NS} (0.59)	10597.7 ** (<.0001)	81.56 ** (<.0001)	376.22 ** (<.0001)	0.46 ^{NS} (0.70)	302.21 ** (<.0001)	95.36 ** (<.0001)
**Error**	4.35	7.60	0.65	1.72	0.01	1.36	2.47	5.64	0.12	3.40	2.88	0.28	1.57
**CV**	1.86	3.47	7.45	5.47	8.50	5.25	6.03	3.43	6.15	5.31	2.35	2.97	5.19
**CD tests @ 5%**	6.43	8.49	2.48	4.05	0.16	3.59	2.22	7.32	1.09	5.68	5.23	1.62	3.86
**CD Checks vs Tests @ 5%**	6.43	8.49	2.48	4.05	0.16	3.59	2.22	7.32	1.09	5.68	5.23	1.62	3.86
**Mean**	112.42	78.51	11.65	24.07	1.35	21.72	26.17	81.84	6.86	37.09	72.05	15.56	25.35
**Standard Error**	1.38	1.37	0.38	0.52	0.04	0.30	0.30	4.62	0.41	1.10	0.99	0.91	0.69
**Minimum**	98.25	67.67	9.40	20.33	1.15	19.90	23.62	42.84	3.45	27.11	65.34	12.44	20.66
**Maximum**	125.00	90.94	14.12	28.21	1.70	24.20	29.07	93.80	8.12	42.63	77.58	24.60	30.13

NS: Non significant; ****** Highly significant; ***** Significant; Values under the parenthesis indicate the Probability value (Pr > F); Alphabets in the superscripts indicate LSD grouping. The BC_2F_3 lines A-13-144-55, A-13-144-53, A-13-144-112, A-13-144-135, A-13-144-139, A-13-144-170 and A-13-144-192 performed equally under normal condition

Supplementary Table 2: Field level screening of *Pup1* Introgressed Improved Samba Mahsuri lines for Bacterial leaf blight (BB) resistance with DRR isolate (DX-020)

Sl. No.	*Pup1*introgressed lines, parent and check	Average lesion length(In cm)	BB score (IRRI-SES)
1	A-13-144-12	< 1	1
2	A-13-144-53	< 1	1
3	A-13-144-55	< 1	1
4	A-13-144-82	< 1	1
5	A-13-144-112	< 1	1
6	A-13-144-135	< 1	1
7	A-13-144-139	< 1	1
8	A-13-144-170	< 1	1
9	A-13-144-192	< 1	1
10	A-13-144-215	< 1	1
11	A-13-144-222	< 1	1
12	A-13-144-223	< 1	1
C_1	Improved Samba Mahsuri	< 1	1
C_2	BPT 5204	18.5±0.54	9

The *Pup1* introgressed BC_2F_3 lines, with all the BB resistance genes showed resistance reaction with BB score 1 similar to recipient parent ISM, while BPT 5204 found susceptible reaction with BB score 9.

Supporting table 1: List of primers sequence, annealing temperature and amplicon size of the molecular markers used in selection for bacterial blight resistance

**Sl no**	**Marker Name**	**Sequence**		**Annealing temperature**	**Amplicon size**	**Reference**
1	pTA 248	F	AGACGCGGGAAGGGTGGTTCCCGGA	55	950/650	Ronald et al 1992
		R	AGACGCGGGTAATCGAAAGATGAAA			
2	xa13 prom	F	GGCCATGGCTCAGTGTTTAT	55	500/250	Sundaram *et al.*, 2011
		R	GAGCTCCAGATCTCCAAATG			
3	xa5FM	SF	GTCTGGAATTTGATCGCGTTCG	55	450&150/450&300	Sundaram *et al.*, 2011
		SR	TGGTAAAGTAGATACCTTATCAAACTGGA			
		RF	AGCTCGCCATTCAAGTTCTTGAG			
		RR	TGACTTGGTTCTCCAAGGCTT			

Supporing table 2: List of parental polymorphic rice SSR markers used in the background survey

Chromosome	Number of polymorphic markers used	Markers name
1	6	RM3148, RM10051, RM10695, RM10963, RM11099, RM315
2	5	RM12434, RM12548 , RM13131, RM13761, RM14102
3	5	RM14311, RM14698, RM14860, RM15185, RM15326, RM15630
4	5	RM16404, RM16495, RM16649, RM16913 RM5511, RM17686
5	5	RM17836, RM3917, RM18222, RM5592, RM19225
6	6	RM19367, RM19660, RM1169, RM20003, RM20378, RM20710
7	5	RM6697, RM21260, RM21539, RM5720, RM21749
8	6	RM22266, RM22554, RM22837, RM6699 RM23350, RM23612
9	5	RM23736, RM23907, RM24199, RM24448, RM24717
10	5	RM25066, RM25147, RM4477, RM25661, RM25181
11	6	RM26225, RM26352, RM26255, RM26829, RM27096, RM27317
12	7 +2*	RM27471, RM27689, RM101, RM27943, , ESSR 20.2, RM28469, RM28827,<br>+<br>RM28011, RM1261*
**Total**	**66+2**	

* Two markers RM28011, RM1261 were utilized for recombinant selection
